# *In Vitro* Biological Performance of Alginate Hydrogel Capsules for Stem Cell Delivery

**DOI:** 10.3389/fbioe.2021.674581

**Published:** 2021-08-27

**Authors:** Jaqueline Brandão de Souza, Gustavo dos Santos Rosa, Mariana Correa Rossi, Fernanda de Castro Stievani, João Pedro Hübbe Pfeifer, André Massahiro Teramoto Krieck, Ana Lívia de Carvalho Bovolato, Carlos Eduardo Fonseca-Alves, Vicente Amigó Borrás, Ana Liz Garcia Alves

**Affiliations:** ^1^ Department of Veterinary Surgery and Animal Reproduction, School of Veterinary Medicine and Animal Science, São Paulo State University, UNESP, Botucatu, Brazil; ^2^ Cell Engineering Lab, Blood Transfusion Center, Botucatu Medical School, São Paulo State University, UNESP, Botucatu, Brazil; ^3^ Institute of Health Sciences, Paulista University-UNIP Bauru, Bauru, Brazil; ^4^ Institut de Tecnologia de Materials, Universitat Politècnica de València, València, Spain

**Keywords:** tridimensional culture, cell encapsulation, biocompatibility, alginic acid, biomaterial

## Abstract

Encapsulation of biological components in hydrogels is a well described method for controlled drug delivery of proteins, tissue engineering and intestinal colonization with beneficial bacteria. Given the potential of tissue engineering in clinical practice, this study aimed to evaluate the feasibility of encapsulation of adipose tissue-derived mesenchymal stem cells (MSCs) of mules in sodium alginate. We evaluated capsule morphology and cell viability, immunophenotype and release after encapsulation. Circular and irregular pores were observed on the hydrogel surface, in which MSCs were present and alive. Capsules demonstrated good capacity of absorption of liquid and cell viability was consistently high through the time points, indicating proper nutrient diffusion. Flow cytometry showed stability of stem cell surface markers, whereas immunohistochemistry revealed the expression of CD44 and absence of MHC-II through 7 days of culture. Stem cell encapsulation in sodium alginate hydrogel is a feasible technique that does not compromise cell viability and preserves their undifferentiated status, becoming a relevant option to further studies of tridimensional culture systems and *in vivo* bioactive agents delivery.

## Introduction

Mesenchymal stem cells (MSCs) have several therapeutic properties due to their immunomodulatory and paracrine actions, being used in a plethora of conditions (Cassano et al., 2018). Isolation of MSCs can be performed in different species and from many tissues, such as bone marrow (Mccarthy et al., 2012), adipose tissue (Yamada et al., 2013) and synovial membrane (Lee et al., 2013).

Although allogeneic MSCs can modulate the immune system, the immune recognition can still occur in some cases, leading to cellular and humoral reactions against donor MSCs (Berglund and Schnabel, 2017). Also, despite exhibiting good results due to direct modulation of cell death in the recipient and to their paracrine action (Naji et al., 2019), MSCs efficacy can be affected by the inflammation, especially in acute cases (Rosa et al., 2020), and freely injected MSCs can also migrate to other tissues (Santos et al., 2019). In this sense, MSC encapsulation creates a physical barrier that does not allow crossing of cells or high molecular weight particles—like antibodies and complement factors—but allows the diffusion of nutrients, metabolites and oxygen at the same time (Orive et al., 2003; Hernández et al., 2010; Nafea et al., 2011). Thus, encapsulation is an alternative to protect allogeneic cells from recipient’s immune response and to avoid or delay cell dispersion, turning the paracrine and immunomodulatory effects longer (Teramura and Iwata, 2009; Hernández et al., 2010).

Cell encapsulation or any other method of *in vitro* tridimensional structure like organoids and spheroids can also benefit cell interaction, favoring cell signaling and expression of pluripotency genes when compared to cells injected in suspension after trypsinization of bidimensional culture systems (Hong et al., 2015; Zhou et al., 2017; Vikram Singh et al., 2018). Once encapsulated, cells can be released either by migration or due to dissolution of the biomaterial in the environment (Lee and Mooney, 2012).

Although several studies evaluated the effect of MSCs encapsulated in alginate in different *in vivo* scenarios (Zhang et al., 2015; Vikram Singh et al., 2018), little is known about cell behavior after encapsulation. Additionally, equids are a well accepted experimental model for translational research. Thus, the aim of this study was to characterize alginate capsules morphologically and to evaluate *in vitro* behavior and viability of encapsulated MSCs.

## Materials and Methods

This study was conducted in accordance with the principles of ethics and well-being in animal experimentation and was approved by the Ethics Committee on Animal Use (CEUA) of the School of Veterinary Medicine and Animal Science of UNESP, Brazil (protocol 234/2018).

### Cell Culture

Cryopreserved cells from a biobank were thawed and characterization was performed in a previous research that evaluated the feasibility of isolation of mule MSCs. Cell culture was performed in culture flasks maintained in an incubator at 37°C and 5% CO_2_, according to Yamada et al. (2013) and Carvalho et al. (2013), using Knockout DMEM^®^, 10% Fetal Bovine Serum, 1% L-glutamine, 1% antibiotic-antimycotic solution (penicillin, streptomycin and amphotericin B), 1% essential amino acids and 0.5% non-essential amino acids. All the medium components were obtained from Thermo Fisher Scientific, Grand Island, New York, USA.

### Encapsulation of AdMSCs and Dissolution for Viability Assessment

Cell encapsulation was performed following the technique previously described by Ma et al. (2003). For that, 5 × 10^4^ cells were homogenized in sterile 1.2% low viscosity alginate solution (Aldrich Materials Science Sigma-Aldrich, Milwaukee, USA) with pH adjusted to 7.4. Alginate concentration was chosen based on the study of Ewa-Choy et al. (2017).

The solution was dripped through a 30 G needle (0.30 mm × 13 mm) in Petri dishes containing a 102 mM calcium chloride aqueous solution, at a controlled rate of 40 ml/h and 10 cm of height. Previous tests were performed to determine optimal rate and height, defined when dripping without drop overlap and coalescence occurred. The drops were maintained for 20 min in the CaCl_2_ solution for the ionic cross-linking occurrence. Each well of a 24-well plate received 10 capsules that were maintained for 24 h in culture medium for clearance of the calcium excess and for cell adaptation to the new tridimensional structure.

At each time point the respective capsules were transferred to 15 ml conical tubes, dissolved with sodium citrate and centrifuged for cell count and cell viability assessment using Trypan Blue technique in a hemocytometer, following the technique described by Tennant (1964). Briefly, capsules were dissolved for 10 min at 37°C in 3.2% sodium citrate solution. After centrifugation and resuspension in PBS, cell concentration and viability were calculated in a hemocytometer filled with a 1:1 solution of cells and trypan blue. The remaining cells attached to the well were counted in a microscope (Zeiss Axiovert40CFL, Axiovision, Carl Zeiss, Oberkochen, Germany) to assess cell release from the capsules.

### Characterization of Alginate Capsules and Encapsulated AdMSCs

#### Morphological Analysis of Capsules

Morphological analyses were performed 24 h after capsule formation adapted from Lopes et al. (2017). Briefly, an 1:20 aqueous solution of toluidine blue (Sigma-Aldrich, St. Louis, MO) was used as a dying solution to mark the capsules by immersion for 5 min. Images were obtained with the Olympus SDF Plapo 1XPF Japan/Olympus SZX16 magnifier (Olympus, Tokyo, Japan).

Mean initial size of the capsules was obtained by Digital Color Camera for Highest-resolution Photomicrography (Leica DFC500 Microsystems, Germany), in 2.5x objective and 1.0x ocular, measuring two perpendicular diameters crossing the center of all capsules. Alginate porosity and analysis of the visible cells on the hydrogel’s surface were assessed by Scanning Electron Microscope (SEM) (FEI Company, Quanta 200), in samples fixed in 10% formaldehyde for 1h, dehydrated and dried (Diniz et al., 2016). Measurements were performed using ImageJ (Java, Scion Corporation, National Institute of Mental Health, Bethesda, Maryland, USA).

### Swelling Behavior

Analysis of the swelling behavior (SB) was conducted gravimetrically based on Hanna et al. (2013) and Bajpai and Kirar (2016) to evaluate the potential of nutrient transportation across the capsule membrane. Two groups were evaluated: capsules alone (*n* = 10) and encapsulated cells (*n* = 10). An overnight post-hydration triplicate was also additionally evaluated in order to verify the capsule behavior and its absorption capability over an extended period.

Briefly, previously weighed capsules were immersed in culture medium at 37°C. Capsules were removed at regular intervals (hourly for up to 7 h), wiped to remove the excess of liquid content, and weighed in a semi-analytical scale (AUY220 scale 0.1 mg, Shimadzu Scientific Instruments Incorporated, Kyoto, Japan). After the measurement, capsules were placed again in the medium. The percentage of absorption was determined using the expression below, where ***M0*** and ***Mt*** are the initial mass and the mass at time “t,” respectively. The measurements were performed in triplicate and mean values were obtained.

$$Swelling\;behavior=\frac{\left(MT-M0\right)}{\left(100\times M0\right)}$$ (1)

Moisture content (Xp%) was determined according to the Association of Official Analytical Chemists (Association of Official Analytical Chemists (AOAC), 2016), with adaptation to the capsule size. A portion of Xg = 5 capsules was maintained at 50°C for 3 h. The capsules were weighed and the procedure of dehydration was repeated till a constant weight was obtained. Since cells would not survive to these steps, Xp% was determined using only naïve capsules. The Xp% was obtained using the equation below, where “*cw*” means the constant weight obtained after dehydration. The tests were performed in triplicate. After dehydration, the capsules were re-hydrated and their final mass was assessed post-overnight.

$$Xp\left(\%\right)=\left[\frac{5-cw}{5}\right]x100$$ (2)

### Encapsulated Cell Analyses

Cell integrity was observed in a Confocal Laser Scanning Microscope (CLSM - TCS SP5, Leica), using the HeNe 633 nm laser wavelength to excite the Qtracker and the 405 nm laser for the 4′,6-diamidino-2-phenylindole (DAPI), to excite and detect the fluorescence of the encapsulated cell sample, according to the technique described by Santos et al. (2019). The whole capsules were placed in excavated slide blanks and observed by CLSM in 2 µm cuts in a 20x objective.

Cell immunophenotype of CD44 and MHC class II was observed by immunohistochemistry. In brief, 4 µm sections were obtained and stretched on histologically charged glass slides (Immunoslide, EasyPath, São Paulo, Brazil) and antigen retrieval was performed using citrate buffer (pH 6) in a pressure cooker (Pascal, Dako, Carpinteria CA, USA). Primary antibodies against CD44 (AbD Serotec, Kidlington, Oxford, UK) and MHC class II mouse anti-horse (AbD Serotec, Kidlington, Oxford, UK) were used. The primary concentration was 1:50 for both antibodies. The polymer detection system (Envision, Dako, USA) was used as the secondary antibody and the sample was incubated at 27°C for 1 h. The molecule 3,3′-diaminobenzidine (DAB) was used as a chromogen for 5 min and then the slides were immersed in Harris-Hematoxylin for 2 min for nuclei staining. Slides were observed in the Confocal Zeiss LSM 510 microscope. The entire section was evaluated to determine CD44 and MHC-II expression, considering either positive or negative labelling. All images were obtained by cameras attached to the microscope.

Cell migration and viability were assessed in triplicate at time points 0, 24, 48, 72 h and 7 days. Cell migration from the capsules to the plastic was determined by direct counting of attached cells, while concentration and viability of encapsulated cells per well were obtained after capsule dissolution in sodium citrate. In order to evaluate the expression of surface markers of AdMSCs after encapsulation, cells were submitted to immunophenotyping according to Carvalho et al. (2014) and Santos et al. (2018). Flow cytometry was performed 24 h and 7 days after encapsulation to assess the behavior of AdMSCs and possible phenotypic changes due to encapsulation. For that, capsules were dissolved using sodium citrate before incubation with the antibodies, and then analyzed by FACS Calibur cytometer (BD, San Jose, CA, USA), as described above.

Metabolic activity was assessed by colorimetric cytotoxicity assay using 3-(4,5-dimethylthiazol-2yl)-2,5-diphenyltetrazolium bromide (MTT) salt, adapted from Uludag and Sefton (1993). Evaluations were made at 0, 24, 72 h and 7 days. Analysis of MTT in adherent cells cultured in monolayers was used as control. In brief, on the day of analysis the conventional medium was replaced with 300 µl of MTT solution (5 mg/ml) and cells were incubated for 3 h at 37°C in the dark. After that, MTT was replaced with isopropyl alcohol to dissolve the crystals formed and the 96-well plate was read. Absorbance was measured at 570 nm using a BioTeK PowerWave XS2 microplate scanning spectrophotometer (Highland Park, Winooski, VT, USA), Gen5 software.

### Statistical Analysis

Experimental data are reported as mean ± SD. The SD was also used as an error bar in the figures. After the descriptive analysis of the data, they underwent a normality test and Dunn’s test was applied for multiple comparisons. The *t*-test or Mann-Whitney was used for double comparisons. Differences were considered statistically significant when *p*-value was <0.05. The software used for all analyses was GraphPad Prism v.7 (GraphPad, San Diego, CA, USA).

## Results

### Cell Encapsulation and Morphological Analysis

Encapsulation using a continuous infusion pump generated approximately 120 capsules/ml of solution dripped in calcium chloride.

**FIGURE 1:**
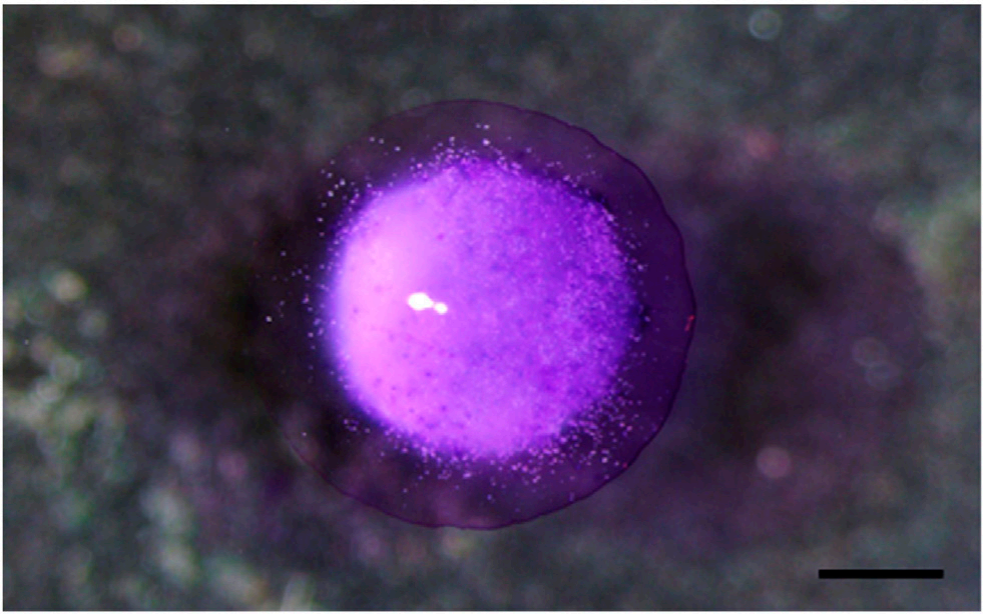
Observation of a capsule by a magnifier, showing a central concentration of cells compared to the edges. Scale bar: 1000 µm.

Capsule observation through the magnifier showed higher cell concentration in the core of the capsule and lower concentration close to the hydrogel margins (Figure 1). Measurement of capsules was performed before and after immersion in toluidine blue solution. Mean initial perpendicular diameters of capsules (D1 and D2) were 1,459 and 1,596 μm, respectively. After immersion in 1:20 toluidine blue solution, the following perpendicular diameters were obtained: D1 = 2,610 ± 40 µm and D2: 2,660 ± 5 µm.

**FIGURE 2:**
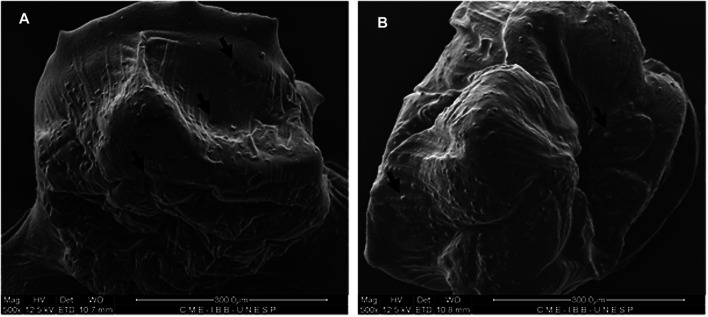
SEM analysis of capsules with MSCs showing the capsule surface, pores and superficial MSCs. Black arrows indicate pores **(A)** and MSCs entrapped in the alginate network **(B)**. Magnification of 500x.

**FIGURE 3:**
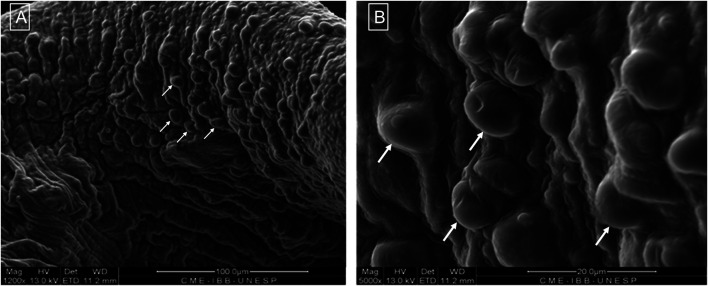
Morphological structure by SEM showing the capsule surface and presence of cells (arrows) at magnification of 1,200x **(A)** and 5,000x **(B)**.

The structure of the microcapsules observed by SEM is shown in Figures 2A,B, 3. Different pore morphologies and measurements were observed, from circular morphology (100–165 µm) to irregular (20–45 µm).

**FIGURE 4:**
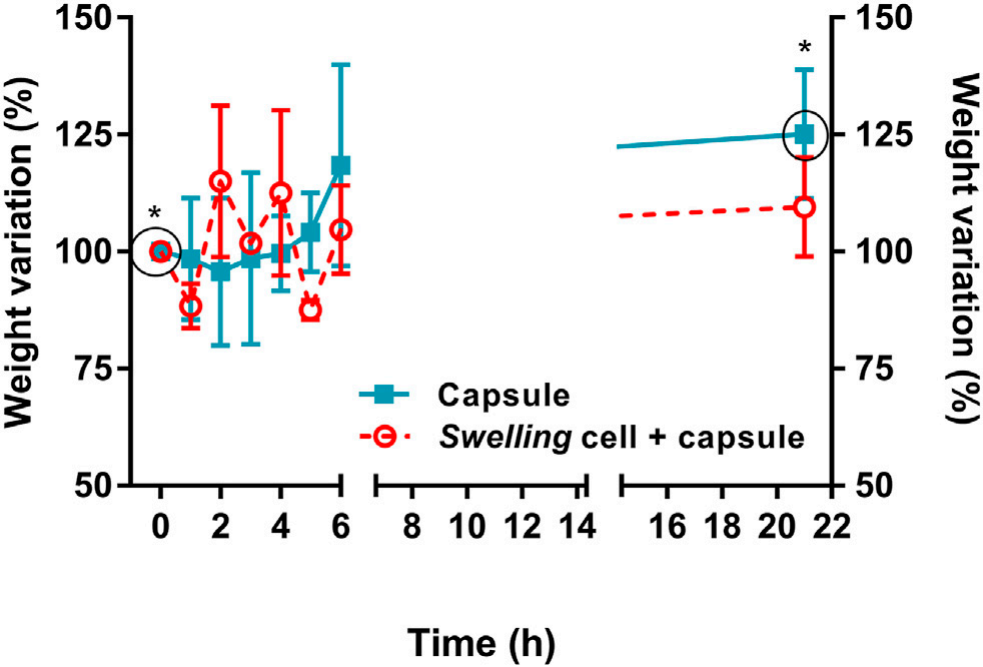
Swelling behavior of pristine alginate capsules (*n* = 10) and capsules with MSCs (*n* = 10). Although capsules with MSCs showed less stability in mass through time, there was a significant increase in mass of the group of capsules alone at 21 h compared to the initial time point (* indicates *p* < 0.05).

Weight variation (%) of mass measurements from the swelling behavior test are shown in Figure 4. Although mass oscillation was more evident in the group with capsules and MSCs, there was a significant increase in mass of the group of capsules at 21 h compared to the initial time point (*p* < 0.05).

**FIGURE 5:**
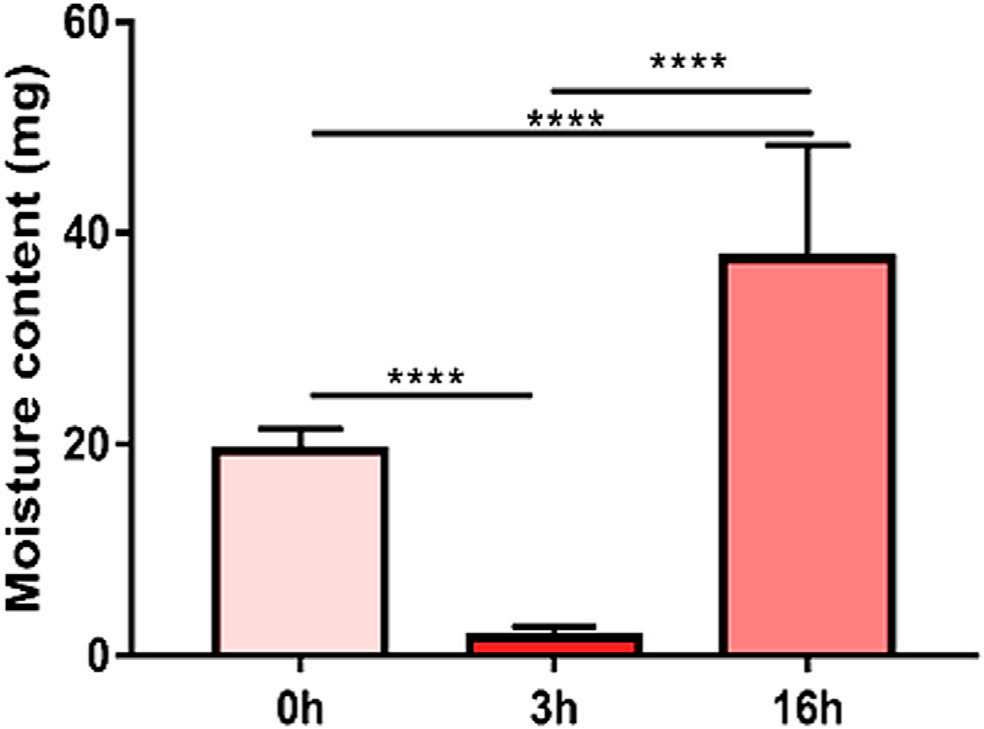
Moisture content of the capsules (*n* = 5). There was a pronounced reduction in moisture content after drying the material at 50°C for 3 h (**** means *p* < 0.0001).

Reduction in moisture content was evident after the drying process (*p* < 0.0001) (90.75 ± 6.26%). Only the group of capsules without cells could be evaluated, since the temperature to which they were subjected aimed the evaluation of the biomaterial *per se* and not the cells, as observed in Figure 5. Capsules showed a good absorption potential after being immersed in culture medium overnight. However, capsules showed a structural modification under direct visualization, acquiring a softer aspect between 72 h and 7 days of culture.

### Cell Integrity and Viability

**FIGURE 6:**
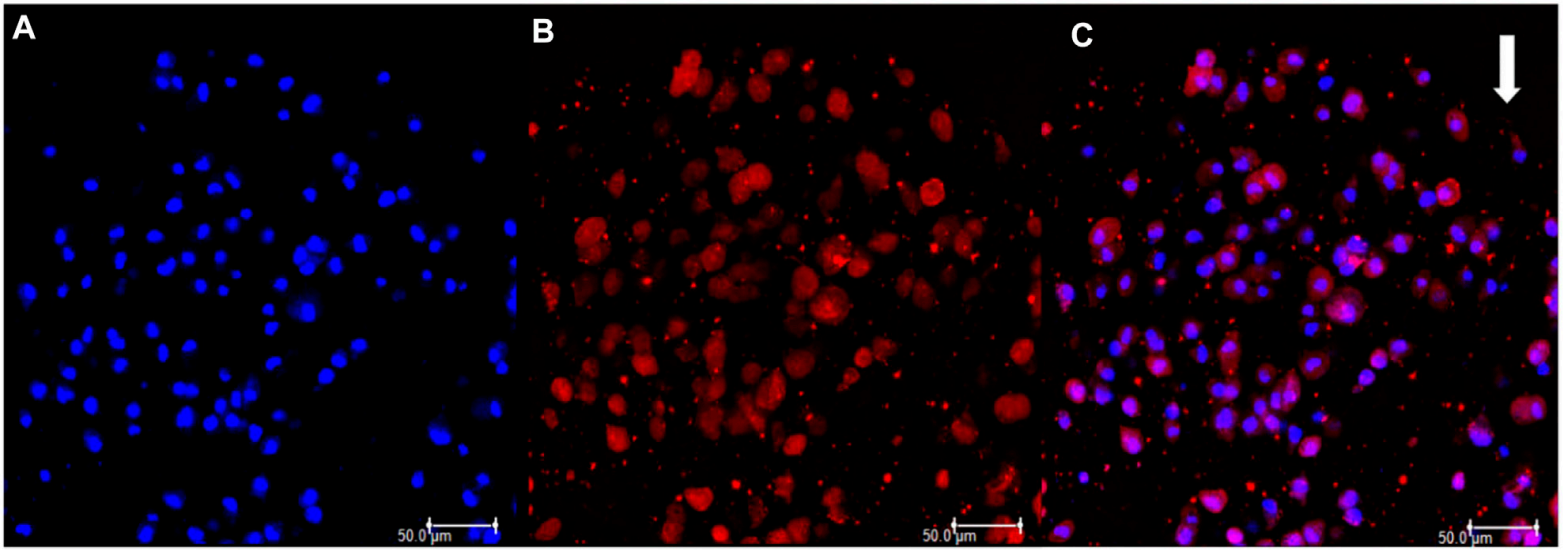
**(A)** DAPI positive labelling showing cell nuclei stained in blue. **(B)** Qtracker positive labeling showing cell cytoplasm stained in red. **(C)** Labellings merged. White arrow indicates the border of the capsule.

The sample marked with the Qtracker and DAPI was observed by Confocal Laser Scanning Microscopy, showing cell membrane and nuclei integrity, confirming cell viability after encapsulation (Figure 6).

**FIGURE 7:**
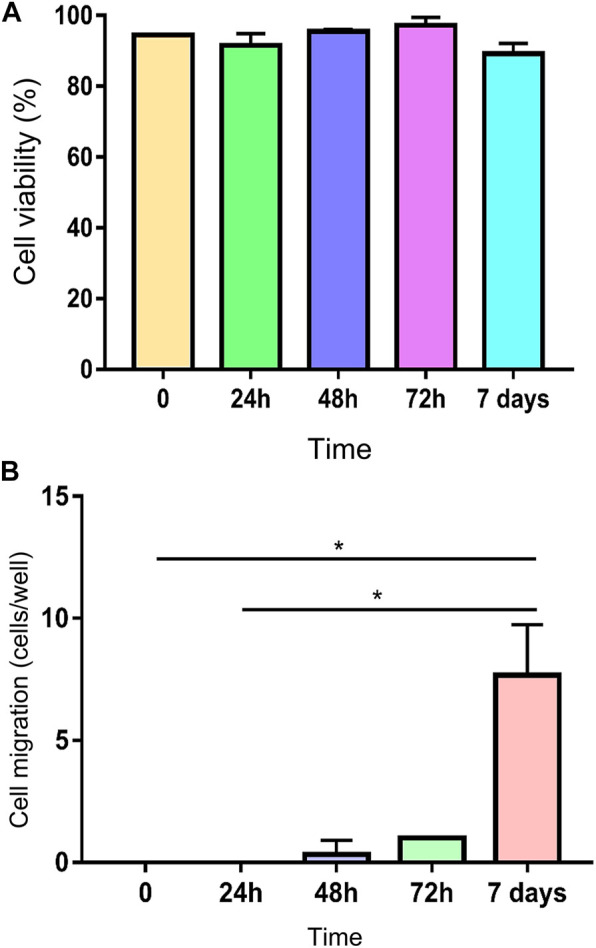
**(A)** Viability of encapsulated MSCs at 0, 24, 48, 72 h and 7 days, showing a maintenance of high viability through time. **(B)** Cell migration from the scaffold to the plastic bottom of the wells (* means *p* = 0.0434).

Encapsulated cells maintained mean viability of 93.57 ± 3.53% (Figure 7A). Cell migration from the scaffolds to the plastic bottom started after 48 h of evaluation, becoming more evident in 7 days with a significant increase of almost 10x compared to the two initial moments (*p* = 0.0434) (Figure 7B).

**FIGURE 8:**
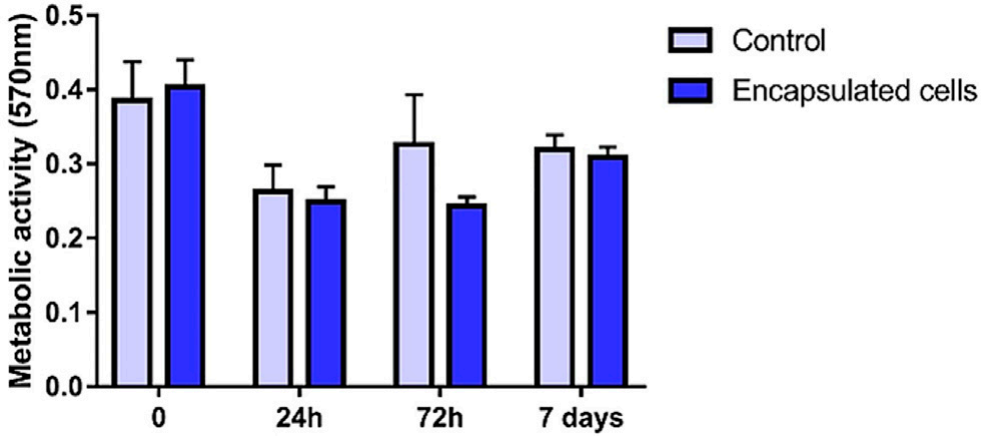
MTT assay showing a decrease in metabolic activity at 24 and 72 h time points and a recovery at 7 days. Adherent MSC culture was used as control.

Despite the absence of statistical difference, MTT assay showed a decline of metabolic activity of encapsulated cells 24 h after encapsulation. Although there was an initial decrease in MTT, subsequent evaluations showed a recovery through time (Figure 8).

### Immunophenotyping and Immunohistochemistry

**FIGURE 9:**
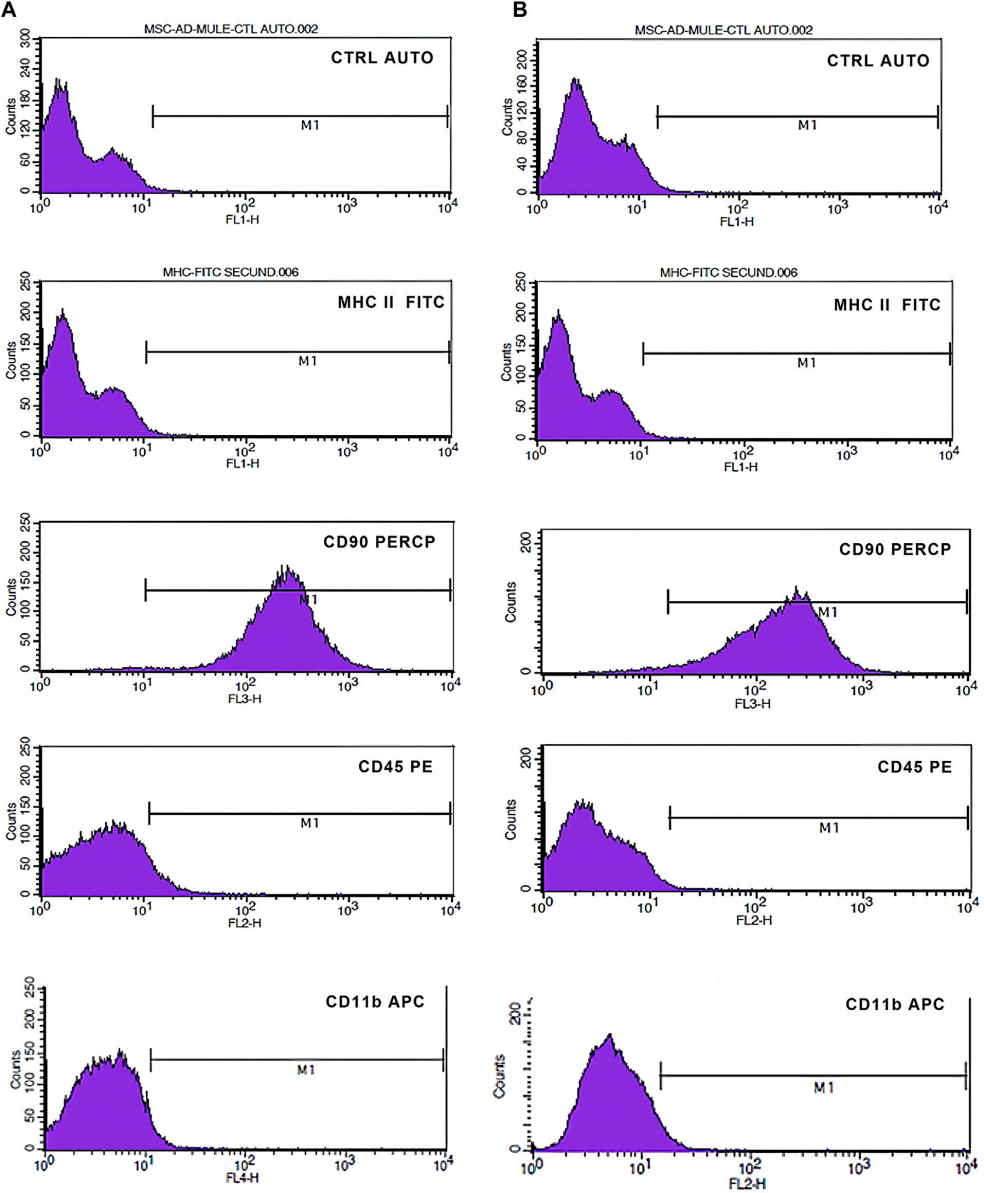
Immunophenotypic characterization by flow cytometry of MSCs 24 h **(A)** and 7 days **(B)** after encapsulation. M1 indicates the area of positivity for each marker. There was maintenance of positivity for CD90 and negativity for CD11b, CD45 and MHCII.

Immunophenotypic profile revealed negativity for CD11b, CD45 and MHC class II and positivity for CD90 at initial time point and after 7 days (Figure 9).

**FIGURE 10:**
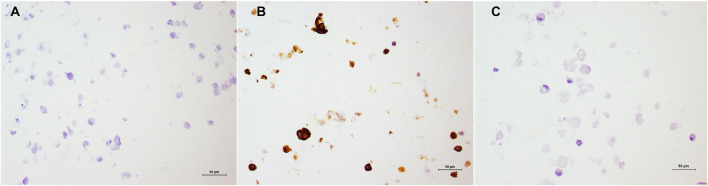
Immunohistochemistry of encapsulated MSCs. **(A)** Negative control of the sample; **(B)** Positive labelling of CD44 and C) Absence of labelling by MHC II. Harris’ hematoxylin counterstaining, 20x.

Immunohistochemistry labeling of the encapsulated cells did not show marking in the negative control (Figure 10A). Cells showed strong labeling for CD44 (Figure 10B) and no expression of MHC II (Figure 10C). Thus, cells were classified into (CD44 +++)/(MHC class II-).

## Discussion

Although MSCs are widely used in different injuries, several factors can interfere in their action, especially in their viability and availability. Thus, systems capable of delivering and maintaining cells at the site of injection are extremely desirable. In this sense, cell encapsulation is a promising alternative (Gao et al., 2019). Alginate is an anionic hydrosoluble polymer extracted from brown algae and some bacteria (*Pseudomonas and Azotobacter*) (Araujo et al., 2013; Hay et al., 2013; Mori et al., 2014; Bajpai and Kirar, 2016), cited as a good polymer option for MSCs encapsulation without cell adhesion due to its biocompatibility, biodegradability and low toxicity (Lee and Mooney, 2012).

In our study, the microcapsule morphometry showed an average initial size of 1,596 µm × 1,459 µm. After staining with toluidine blue (1:20), we observed an increase in the dimensions of the microcapsules due to the absorption capacity, which was confirmed by swelling assay and post-hydration moisture content evaluation. The initial size stability of the capsules can be explained by the elasticity force of the hydrogel that acts against osmotic force by stretching the biomaterial network, generating a balance of forces that prevents its deformation (Ganji et al., 2010). However, since initial stability was not linear in the group with MSCs, we postulate that cellular activity may have a role in the interaction between the hydrogel and the medium. Unfortunately, this interaction was not evaluated meticulously, being a limiting factor of the study.

Moisture content refers to the percentage water in relation to dry weight (Lopes et al., 2017). The reduction in moisture content was evident (90.75 ± 6.26%), corroborating the results of Lopes et al. (2017), which observed a content of 97.70 ± 0.03%. After dehydrating the biomaterial and immersing it again in culture medium for 12 h, we observed a potential for recovery and absorption. According to Herrero et al. (2007), this is an important signal of proper nutrient and oxygen delivery, since it shows the capacity of diffusion of the medium through the biomaterial.

The increase in capsule size and soft aspect over time may be explained by the occurrence of disintegration of the biomaterial due to its interaction with the culture medium. According to Bajpai and Kirar (2016) and Kikuchi et al. (1999), the ions of the medium enter the capsule network and promote ion exchange, which causes a relaxation of the M-chains of the alginate structure and creates spaces within the network, increasing water absorption. The fully hydrated structure begins to lose its structural integrity due to the rupture of the cavities and the alginate chains start to disintegrate and dissolve, generating the soft aspect observed macroscopically.

Evaluation of pore diameter and morphology was performed by SEM. Pores were either morphologically circular, with 100–165 μm, or irregular with 20–45 µm. Scaffolds with pores between 250 and 500 µm lead to extracellular matrix production by chondrocytes, while pores of 50–200 µm resulted in undifferentiated cells (Lien et al., 2009). As the pore size becomes larger, the rate of cell growth and the glycosaminoglycan production increases, as well as the expression of genetic markers for aggrecan and type I, type II and type X collagens. Even though the dehydration process necessary to the SEM preparation may have altered the pore structure (Aston et al., 2016), the expression of stem cell surface markers was preserved after 7 days of culture in a scaffold with pores ranging from 20 to 165 μm, which demonstrates maintenance of an undifferentiated state, corroborating the study of Lien et al. (2009).

Lang et al. (2017) demonstrated decrease in cell viability after injection through a 25 G needle compared to 20 G. According to the same study, there is a natural variation in size of cells in the same organism, so larger stem cells are more likely to be cut after passing through small diameters. However, in this study the use of a 30 G needle did not cause significant impairment of cell membrane integrity, as demonstrated by fluorescence microscopy, nor their viability, as observed by trypan blue dye exclusion method. We deduce that the higher viscosity of alginate, compared to more liquid solutions such as PBS, might protect the cells by creating an alginate cover layer that avoids cell clustering and smashing during the dripping process through the needle.

Based on cell viability through time, our results demonstrated an adequate cell concentration, corroborating the results of Ma et al. (2003), despite the difference in capsule size between both studies (1,500 μm vs. 3,400 mm). Although calcium toxicity was not evaluated in this study, capsules were maintained in culture media for 24 h prior to the tests, to eliminate the excess of calcium chloride accumulated during the encapsulation process that could promote some degree of toxicity (Ma et al., 2003). Cell viability was constantly high through the 7 days, corroborating the results of human cells demonstrated by Ma et al. (2003), showing cell viability higher than 90% for up to 4 weeks.

However, initial decrease in MTT demonstrates that the encapsulation process is not absolutely innocuous. Results of MTT indicate that although a negative effect in cell metabolism occurs at first, it is not sufficient to cause expressive cell death, once cell viability was maintained high through all time points.

Cell migration from the scaffold to the suspension medium and subsequent cell adherence to the plastic confirmed the active metabolic status of the MSCs, since they demonstrated attachment to the plastic bottle and cytoskeleton modification, adopting fibroblastoid morphology.

Although *in vitro* studies still can not mimetize the exact events that occur *in vivo,* they contribute to the comprehension of cell-cell and cell-environment interactions, which generates safer *in vivo* studies (Moshtagh et al., 2020).

## Conclusion

Stem cell encapsulation using a continuous infusion pump system was a successful and feasible method. The constant flow is highly desirable in order to maintain homogeneity of the capsules and, consequently, reliability of results. The present study considered several assays that, taken together, confirmed that cell encapsulation of mule mesenchymal stem cells is a viable technique that demonstrates potential for *in vivo* applications. Structural characterization of pores and morphology showed proper nutrient diffusion through the material, which is imperative for the maintenance of cell integrity, metabolism and viability. Since MSCs immunophenotypic properties were conserved through time, our results substantiate further *in vitro* and *in vivo* studies aiming to evaluate MSCs behavior.

## Data Availability

The raw data supporting the conclusion of this article will be made available by the authors, without undue reservation.
